# Marine Collagen Peptide Fraction from *Lutjanus erythropterus* Scales: A Multifunctional Bioactive for Intestinal Barrier Protection and Redox Modulation in Ulcerative Colitis

**DOI:** 10.3390/foods15142480

**Published:** 2026-07-13

**Authors:** Qi Deng, Muhammad Kashif Imtiaz, Jiabao Huang, Ali Imran, Mei Qiu, Zhijiia Fang, Rui-Bo Jia

**Affiliations:** 1 Guangdong Provincial Key Laboratory of Aquatic Product Processing and Safety, Guangdong Provincial Engineering Technology Research Center of Seafood, Guangdong Province Engineering Laboratory for Marine Biological Products, Key Laboratory of Advanced Processing of Aquatic Product of Guangdong Higher Education Institution, College of Food Science and Technology, Guangdong Ocean University, Zhanjiang 524088, China; dengqi@gdou.edu.cn (Q.D.); kashif3385@gmail.com (M.K.I.); 15361167540@163.com (J.H.); qiumeigdou@163.com (M.Q.); fangzhijia@gdou.edu.cn (Z.F.); 2 Collaborative Innovation Center of Seafood Deep Processing, Dalian Polytechnic University, Dalian 116034, China; 3 Department of Food Science, Faculty of Life Sciences, Government College University Faisalabad, Faisalabad 38000, Punjab, Pakistan; dr.aliimran@gcuf.edu.pk

**Keywords:** collagen peptides, ulcerative colitis, oxidative stress, gut microbiota

## Abstract

Low-molecular-weight collagen peptides from food processing byproducts offer a sustainable approach to mitigating intestinal inflammation, yet their mechanistic roles remain incompletely understood. We evaluated red fish scale collagen peptides-I (LSCP-I), a <3 kDa collagen peptide fraction derived from *Lutjanus erythropterus* scales, in cellular and murine models of colitis. In Caco-2 cells subjected to macrophage-mediated inflammatory injury, LSCP-I (50 µg/mL) increased proliferation by 35%, enhanced migration by 40%, preserved barrier integrity, reduced reactive oxygen species (ROS) by 45%, decreased lipid peroxidation by 30%, and restored glutathione (GSH) and superoxide dismutase (SOD) activity. In mice with dextran sulfate sodium (DSS)-induced colitis, oral administration of LSCP-I at 200, 400 and 800 mg/kg/day attenuated weight loss and diarrhea, lowered intestinal permeability by 38%, enhanced colon histology, and restored the balance between pro- and anti-inflammatory cytokines. Mechanistically, LSCP-I activated the Nrf2 antioxidant pathway and partially restored gut microbiota composition. These results demonstrate that LSCP-I reinforces intestinal barrier function, restores redox homeostasis, and modulates host–microbiota interactions, establishing its potential as a functional food ingredient for the prevention and management of inflammatory bowel disease.

## 1. Introduction

Ulcerative colitis (UC) is a chronic relapsing inflammatory disorder of the colon characterized by epithelial barrier dysfunction, dysregulated immune responses, and oxidative stress [1]. The global incidence of UC and related inflammatory bowel diseases has steadily increased, imposing substantial burdens on public health and quality of life [2,3]. The intestinal epithelium, a highly dynamic barrier that regulates nutrient absorption while preventing pathogen and toxin translocation, is central to this pathology [4]. Together, these factors drive mucosal injury and clinical symptoms including weight loss, diarrhea, and rectal bleeding. The global incidence of UC and related inflammatory bowel diseases has steadily increased, imposing substantial burdens on public health and quality of life. Although conventional therapies including aminosalicylates, corticosteroids, and biologics can induce remission, treatment resistance, adverse effects, and long-term intolerance remain significant challenges. These limitations have motivated interest in dietary strategies that modulate colonic inflammation and barrier integrity with improved safety profiles. The intestinal epithelium, a highly dynamic barrier that regulates nutrient absorption while preventing pathogen and toxin translocation, is central to this pathology. Disruption of this barrier is a hallmark of UC and is closely associated with oxidative stress-mediated epithelial injury. Excessive reactive oxygen species (ROS) production impairs tight junction integrity, alters gut microbiota composition, and triggers inflammatory cascades, ultimately compromising intestinal homeostasis [5,6,7]. Oxidative stress has thus emerged as a central driver of epithelial dysfunction, linking environmental and dietary stressors to intestinal pathology through mechanisms involving apoptosis, autophagy, and redox imbalance [5].

Bioactive peptides derived from food proteins have emerged as promising dietary modulators of intestinal health because of their antioxidant, anti-inflammatory, and barrier-protective properties [8,9]. Among these, marine collagen-derived peptides have attracted increasing attention due to their favorable amino acid composition, high digestibility, low antigenicity, and sustainable origin from fish processing byproducts [10,11]. In particular, collagen peptides enriched in glycine, proline, hydroxyproline, and hydrophobic amino acids exhibit strong radical scavenging activity and may contribute to epithelial repair, redox regulation, and intestinal homeostasis [10,12,13]. Recent studies further demonstrated that some marine collagen peptide systems can attenuate DSS-induced colitis by restoring intestinal barrier integrity, modulating gut microbiota composition, and suppressing oxidative stress-associated inflammatory responses [14,15,16].

Increasing evidence indicates that the biological activity of food-derived peptides is strongly influenced by molecular weight distribution. Compared with higher-molecular-weight hydrolysates, low-molecular-weight peptide fractions (<3 kDa) exhibit improved solubility, intestinal transport efficiency, and cellular accessibility, enabling more effective interaction with intracellular antioxidant and inflammatory signaling pathways [10,12,17]. Unlike isolated single peptides, naturally occurring peptide combinations may exert synergistic or complementary biological effects because of their structural diversity and multifunctional modes of action [14,18]. Such low-molecular-weight peptide fractions therefore more closely reflect the physiological complexity of dietary peptide exposure following gastrointestinal digestion and food processing [17,19]. Nevertheless, despite growing interest in peptide-based interventions for intestinal inflammation, the mechanistic roles of marine collagen-derived low-molecular-weight peptide fractions in regulating oxidative stress and epithelial barrier dysfunction during ulcerative colitis remain insufficiently understood.

Oxidative stress is now recognized as a critical contributor to intestinal epithelial injury and barrier disruption in ulcerative colitis [1,5]. Excessive reactive oxygen species accumulation impairs tight junction proteins, disrupts gut microbial homeostasis, and activates inflammatory signaling cascades, thereby exacerbating mucosal damage and disease progression [5,20,21]. Cellular defense against oxidative injury is primarily regulated through the KEAP1-Nrf2 signaling pathway, which coordinates the transcription of multiple antioxidants and cytoprotective enzymes, including HO-1, SOD, and glutathione-associated systems [21,22,23]. Several food-derived peptide systems, including wheat, rice, oat, and marine-derived peptides, have been reported to alleviate DSS-induced colitis through activation of Nrf2-mediated antioxidant responses and restoration of intestinal barrier function [15,24,25,26]. Among these, LSCP-I possesses several distinctive characteristics, which include high levels of glycine, proline, and hydroxyproline residues, excellent digestibility, favorable biocompatibility, and a structural composition closely associated with extracellular matrix maintenance and tissue repair [10,11,12,13,18]. Furthermore, low-molecular-weight marine collagen peptide fractions (<3 kDa) exhibit enhanced intestinal absorption and bioavailability, enabling more efficient interaction with intracellular redox-sensitive signaling pathways than larger peptide populations [10,11,12,13]. However, despite emerging evidence supporting the anti-colitic activity of marine collagen peptides, it remains unclear whether low-molecular-weight marine collagen peptide ensembles can coordinately regulate oxidative stress and intestinal barrier integrity through KEAP1-Nrf2-associated mechanisms, and the molecular basis underlying these effects remains poorly defined. Compared with previously reported marine collagen peptides, LSCP-I is derived from *Lutjanus erythropterus* scales, an underutilized fish processing byproduct with potential value in sustainable marine resource utilization. The novelty of the present study lies in the evaluation of a low-molecular-weight peptide-enriched fraction from this specific source and its integrated assessment across epithelial barrier function, oxidative stress regulation, KEAP1-Nrf2 signaling, inflammatory cytokine modulation, and gut microbiota-associated responses in DSS-induced colitis.

This study aimed to evaluate the protective effects of LSCP-I, a <3 kDa collagen peptide fraction obtained from *Lutjanus erythropterus* scales, against oxidative stress-induced intestinal epithelial injury and dextran sulfate sodium (DSS)-induced colitis. Using both intestinal epithelial cell models and a murine colitis model, we sought to determine whether LSCP-I could (i) attenuate oxidative damage, (ii) restore intestinal barrier function, and (iii) activate KEAP1-Nrf2-mediated antioxidant defenses. By integrating cellular and in vivo approaches, this study provides mechanistic insight into the role of marine collagen-derived low-molecular-weight peptide fractions in regulating redox homeostasis and intestinal barrier function during ulcerative colitis, thereby advancing current understanding of marine peptide-based strategies for intestinal inflammatory disorders.

## 2. Materials and Methods

### 2.1. Preparation of LSCP-I

Scales of *Lutjanus erythropterus* were obtained from a local aquatic processing facility, thoroughly washed with distilled water to remove adhering impurities, and stored at −20 °C until use. Hydrolysis was conducted using a composite protease with a declared activity of 120 U mg^−1^ (Shanghai Yuanye Bio-Technology Co., Ltd., Shanghai, China). The reaction was performed at pH 5.93 and 54.03 °C for 2.26 h with an enzyme dosage of 100 U mL^−1^. The substrate concentration was 1% (*w*/*v*) and the enzyme-to-substrate ratio was 100 U mL^−1^: 1% (*w*/*v*) (equivalent to 10 U/mg substrate). These conditions were selected based on preliminary processing trials and literature-reported ranges for marine collagen peptide production [27,28,29,30]. The degree of hydrolysis was not determined in the present study, as the primary objective was biological evaluation of the LSCP-I fraction rather than optimization of hydrolysis parameters. After hydrolysis, the enzyme was inactivated by boiling for 5 min, followed by centrifugation at 9000 rpm for 30 min at 4 °C. The supernatant was freeze-dried and designated as red fish scale collagen peptides (LSCPs).

To obtain peptide fractions with different molecular weight distributions, LSCPs were sequentially fractionated using ultrafiltration membranes (Millipore, Burlington, MA, USA) with molecular weight cut-off values of 8 and 3 kDa. Three fractions were obtained: LSCP-I (<3 kDa), LSCP-II (3–8 kDa), and LSCP-III (>8 kDa). Ultrafiltration-based fractionation is widely used for the enrichment of low-molecular-weight antioxidant peptides from marine collagen hydrolysates because peptide bioactivity, intestinal transport, and cellular accessibility are strongly influenced by molecular size. Several studies have reported that peptide fractions below 3 kDa exhibit enhanced antioxidant activity and biological efficacy compared with higher-molecular-weight fractions [27,31,32,33].

LSCP-I was prioritized for biological evaluation because peptide fractions below 3 kDa are widely reported to exhibit improved solubility, intestinal transport, and cellular accessibility compared with higher-molecular-weight fractions [7,9,14].

### 2.2. Experimental Materials and Reagents

Dextran sulfate sodium (DSS; MW 36–50 kDa) was obtained from Beijing Coolaber Technology Co., Ltd. (Beijing, China). Enzyme-linked immunosorbent assay (ELISA) kits for IL-1β (Cat# E-EL-M0037, detection range 15.63–1000 pg/mL, sensitivity 9.38 pg/mL, intra-assay CV < 10%, inter-assay CV < 12%), IL-6 (Cat# E-EL-M0044, detection range 31.25–2000 pg/mL, sensitivity 18.75 pg/mL), IL-10 (Cat# E-EL-M0046, detection range 15.63–1000 pg/mL, sensitivity 9.38 pg/mL), and TNF-α (Cat# E-EL-M3063, detection range 7.81–500 pg/mL, sensitivity 4.69 pg/mL) were sourced from Elabscience Biotechnology Inc., (Wuhan, China). Oxidative stress assay kits for malondialdehyde (MDA; Cat# AKFA013M), superoxide dismutase (SOD; Cat# AKAO001M-50S), and glutathione (GSH; Cat# AKPR008M) were purchased from Boxbio Biotechnology (Beijing, China). Fluorescein isothiocyanate dextran (FITC-dextran, 4 kDa) was obtained from Oubei Biotechnology (Shaanxi, China), and D-lactate (D-LA) detection kits were obtained from Suzhou Greis Biotechnology Co., Ltd. (Suzhou, China).

Primary antibodies against occludin (Cat# AF7644, rabbit monoclonal, dilution 1:1000), JAM-1 (rabbit monoclonal, dilution 1:1000), Nrf2 (Cat# AF1609, rabbit monoclonal, dilution 1:1000), and β-actin (Cat# AF5001, mouse monoclonal, dilution 1:5000), along with RIPA lysis buffer and BCA protein assay kits, were purchased from Beyotime Biotechnology (Shanghai, China). Lipopolysaccharide (LPS, *Escherichia coli*) was also obtained from Beyotime. Caco-2 cells were acquired from the Cell Bank of the Chinese Academy of Sciences (Shanghai, China). Dulbecco’s modified Eagle’s medium (DMEM, high glucose) and fetal bovine serum (FBS) were purchased from Gibco (Grand Island, NY, USA).

The selection of these reagents and biomarkers follows established protocols for evaluating intestinal inflammation, oxidative stress, and epithelial barrier integrity in marine bioactive peptide studies [10,12].

### 2.3. Cell Culture and Cytotoxicity Assessment

Caco-2 cells between passages 25 and 35 were used for all experiments to minimize passage-related variability in differentiation, transepithelial electrical resistance (TEER) development, and tight junction formation.

To evaluate cytotoxicity, cells were exposed to LSCP-I (50, 100, 200, 400, 800 and 1600 μg/mL) for 24 h. Cell viability was determined using the CCK-8 assay by measuring absorbance at 450 nm. Cell viability (%) was calculated as

$$Cell\;viability\left(\%\right)=\frac{D_{i}-D_{0}}{D_{j}-D_{0}}\times 100$$

Here, *D*_0_ denotes the blank absorbance, *D_i_* corresponds to the sample group absorbance, and *D_j_* refers to the untreated control.

This assay is widely used to evaluate peptide cytocompatibility and proliferative effects in intestinal epithelial models [13].

### 2.4. Construction of the Inflammatory Caco-2 Model

#### 2.4.1. Effect of LPS on the Activity of Caco-2 Cells

Caco-2 was inoculated at a density of 1 × 10^4^ pcs/well on a 96-well plate for 24 h and the medium was aspirated. LPS was dissolved in serum-free DMEM high-glucose medium at 0.1, 0.5, 1, 10, and 100 μg/mL; the LPS-containing medium was added to each well, and LPS-free serum was used as the control group. Cell viability was assessed using CCK-8 to determine direct cytotoxic effects. LPS concentrations of 0.1–100 μg/mL were selected to screen both direct epithelial cytotoxicity and macrophage-conditioned inflammatory injury. A serum-free, LPS-free control was included to distinguish the effect of serum deprivation from LPS-mediated effects.

#### 2.4.2. Macrophage-Conditioned Medium Model

RAW 264.7 cells were cultured in high-glucose DMEM supplemented with 10% fetal bovine serum (FBS) and maintained at 37 °C in a humidified 5% CO_2_ atmosphere. Cells were passaged before reaching over-confluence, and only healthy cells in the logarithmic growth phase were used for conditioned medium experiments. RAW 264.7 was inoculated at 1 × 10^5^/mL in a 6-well plate, LPS was dissolved in a serum-free DMEM high-sugar medium at 0.1, 0.5, 1, 10, and 100 μg/mL, and 2 mL of LPS-containing medium was added per well. The blank group was DMEM high-sugar medium without LPS. After 24 h of incubation of RAW 264.7 cells under serum-free LPS, the medium was taken out as M1 RAW 264.7 cell conditioned medium, which was added to a 96-well plate inoculated with Caco-2; the cell viability was determined after 24 h of incubation, and the optimal LPS stimulation concentration was determined according to the viability of Caco-2 cells. RAW 264.7 cells were stimulated for 2, 4, 8, 12, 16, 20, and 24 h. The 16 h conditioned medium was selected because it induced reproducible inflammatory injury while maintaining sufficient Caco-2 viability for subsequent intervention experiments. The RAW 264.7-conditioned medium model reflects a combined inflammatory microenvironment and may include macrophage-derived cytokines, residual LPS, nutrient depletion, and serum-free stress. Therefore, the model should be interpreted as an inflammatory conditioned medium injury model rather than evidence of a single mediator-specific mechanism. The killing rate against Caco-2 cells is calculated according to the following formula:

$$Caco-2\;injury\;rate\;(\%)=\frac{{D_{j}-D}_{i}}{D_{j}-D_{0}}\times 100$$

where *D_i_* is the absorbance of the experimental group, *D_j_* is the absorbance of the control group, and *D*_0_ is the absorbance of the blank.

This approach models an inflammatory microenvironment using macrophage-derived soluble mediators, consistent with prior work demonstrating collagen peptide attenuation of inflammatory responses in Caco-2 monolayers [12]. Because macrophage polarization markers were not comprehensively quantified, the present study does not claim definitive M1 polarization based solely on morphological changes.

### 2.5. Intestinal Epithelial Barrier Formation and TEER Measurement

Caco-2 cells were seeded in transwell 12-well plates (Corning), and 0.5 mL of DMEM medium containing 10% serum was added to the upper and lower chambers for 21 days; the fluid was changed every two days for 0~7 days, and once a day for 8~21 days. The upper chamber with a resistance value greater than 400 Ω was selected for further experiments. The blank group was supplemented with conditioned medium generated by RAW 264.7 cells without LPS stimulation, the inflammatory injury group was supplemented with conditioned medium generated by RAW 264.7 cells under 1 μg/mL LPS stimulation, and the LSCP-I intervention group received LSCP-I at 400 or 800 μg/mL dissolved in the macrophage-conditioned medium before application to the Caco-2 monolayers. Therefore, the observed effects may involve epithelial protection and/or interaction with inflammatory mediators present in the conditioned medium. The present design does not fully distinguish direct epithelial effects from macrophage-mediated effects.

### 2.6. Scratch Migration Assay

Caco-2 cells were inoculated in a 6-well plate at 1 × 10^4^/mL; after the cells were full, the 200 μL spear tip was used to line the plate three times, the scraped cells were rinsed with DEME medium containing 10% serum, and the width of the scratch was photographed under the microscope. DEME medium was added to the blank group; RAW 264.7 conditioned medium was added to the inflammatory injury group; RAW 264.7 conditioned medium containing 200, 400 and 800 μg/mLL was added to the LSCP-I intervention group; and 800 μg/mL LSCP-I was added to the LSCP-I control group. The healing status of the scratches was observed after 24 h of culture. Because the scratch assay was conducted in serum-containing medium, wound closure may reflect both migration and proliferation.

### 2.7. Oxidative Stress Measurement in Caco-2 Cells

Following migration assays, cells were lysed and the supernatants analyzed for oxidative biomarkers, including SOD activity, GSH content, MDA levels, and intracellular ROS using commercial kits according to the manufacturer’s instructions. Intracellular ROS was additionally quantified by a fluorescence microplate reader after DCFH-DA staining. Cells were incubated with DCFH-DA at a working concentration of 10 μM for 30 min at 37 °C in the dark. Fluorescence was measured at excitation/emission wavelengths of 488/525 nm using a fluorescence microplate reader. Oxidative stress biomarkers (SOD, GSH, MDA) were normalized to total protein content determined by the BCA method.

### 2.8. Animal Experiments (DSS-Induced Colitis Model)

Male C57BL/6 mice, aged 6–7 weeks and weighing between 18 and 22 g, were obtained from Zhuhai Beston Biotechnology Co., Ltd. (Zhuhai, China). The animals were housed under specific pathogen-free conditions, with unrestricted access to standard laboratory chow (GB 14924.3-2010) and sterile distilled water. Mice were housed under a 12 h light/dark cycle at a constant temperature of 22 ± 2 °C with relative humidity maintained at 65–70%. All procedures adhered strictly to the laws, regulations, and ethical guidelines of the People’s Republic of China. The experimental protocol was approved by the Laboratory Animal Care Ethics Committee of the Guangdong Academy of Agricultural Sciences, and the study was performed in the SPF-accredited animal facility of the School of Food Science and Technology, Guangdong Ocean University (license No. SYKX (Guangdong) 2014 0053).

Dextran sulfate sodium (DSS) powder was dissolved in sterile drinking water to prepare a 3% (*w*/*v*) DSS solution. Following one week of acclimatization, the experimental protocol was initiated. Mice received 3% DSS in drinking water for 7 consecutive days to induce acute colitis. LSCP-I was administered daily by oral gavage at 200, 400, or 800 mg/kg body weight in a volume of 100 μL, starting on Day 1 (the same day as DSS initiation) and continuing through Day 7. This design represents a concurrent preventive/intervention model rather than a post-disease therapeutic treatment model. On the initial day of the experiment, mice were randomly allocated to five groups (*n* = 6 per group): a normal control, a DSS model group, and three DSS groups administered LSCP I at low (200 mg/kg), medium (400 mg/kg), or high (800 mg/kg) doses. At study termination, all animals were euthanized; colon length was recorded; and serum, colonic contents, and colon tissue were harvested from each mouse. Tissue samples were either stored at −80 °C or fixed in 4% neutral buffered formalin for subsequent analyses. The DSS model effectively replicates ulcerative colitis-like pathology and has been widely used to evaluate dietary peptide interventions [10].

### 2.9. Disease Activity Index (DAI) Scoring

During the experiment, the clinical progression of colitis was assessed by the disease activity index (DAI) score. During DSS processing, DAI is scored daily according to the criteria in Table 1. Each symptom was scored based on a previous report. Blood in the stool: Use bamboo skewers to pick out a small amount of feces on white porcelain plates, slides or filter paper; add 2~3 drops of o-toluidine glacial acetic acid solution, then add 2~3 drops of hydrogen peroxide solution, and observe the results immediately after mixing. Negative—No discoloration after adding the reagent for 2 min; weak positive—after adding the reagent, the initial light blue gradually changes to blue; positive—light blue at first after adding reagent, gradually showing obvious blue–brown color; strong positive—blue–brown color appears immediately after adding the reagent.

### 2.10. Histological Analysis

To assess the severity of colitis, the distal colon portion was washed with pre-chilled PBS, immediately fixed in 4% neutral buffered formalin for 24 h, and then cut into 5 mm thin slices with a tissue microtome after dehydrated paraffin embedding. Hematoxylin and eosin staining (H&E) was used to detect changes in colon tissue morphology. Slices are viewed using a biological microscope.

### 2.11. Biochemical Analyses in Colon Tissue

The concentrations of IL-1β, IL-6, IL-10 and TNF-α in mouse colon tissue were determined by ELISA kit. The weighed mouse colon tissue was thawed on ice, and according to the requirements of the kit instructions, the colon tissue was fully homogenized with pre-chilled PBS buffer (pH = 7.4) according to the weight-to-volume ratio (*w*/*v*) of 1:9, and then centrifuged (3000 rpm/min; 10 min) to remove the supernatant (take 1 mL in an enzyme-free PE tube). The subsequent measurement steps are strictly followed in accordance with the kit instructions. Cytokine concentrations in colon tissue homogenates were normalized to total protein content determined by the BCA assay and are expressed as pg/mg protein.

### 2.12. Determination of Intestinal Permeability

Intestinal barrier function was assessed using fluorescein isothiocyanate (FITC)-labeled dextran spectrophotometry. After fasting for 12 h, mice were given 400 mg/kg FITC-glucan under dark conditions 4 h before dissection, and the blood of the mice was removed by eye blood sampling under protective light and then placed in a heat-free E-P tube for 1 h and centrifuged at 3000 rpm/min for 10 min; the serum was removed and loaded into a heat-free EP tube. Then, 0.1 mL of serum was taken in a 96-well fluorescence plate under the protection of light, and the fluorescence value (excitation wavelength 485 nm, emission wavelength 520 nm) was detected by a microplate reader and quantified according to the FITC-dextran concentration calibration curve.

Serum levels of D-lactate (D-LA) and lipopolysaccharide (LPS) were measured using commercially available detection kits. All analytical procedures were performed precisely according to the protocols provided by the respective kit manufacturers.

### 2.13. Western Blotting

The preserved colon tissue was removed from the −80 °C refrigerator, a certain amount was weighed in 2 mL of enzyme-free EP tubes at 1:100 (mg/mL), lysate containing 1% PMSF was added to each tube, and 2–3 enzyme-free steel beads were added for tissue homogenization. After colon tissue homogenization was completed, the sample was lysed on ice for 30 min. Following the completion of lysis, the samples were centrifuged at 4 °C and 3000 rpm for 10 min. The resulting supernatant was then collected into 0.5 mL enzyme-free centrifuge tubes. Protein concentrations in the samples were quantified using the BCA assay kit. After adjusting the sample protein concentration to 3.125 mg/mL, SDS-PAGE loading buffer was added according to the instructions and denatured in boiling water for 5 min. Samples were separated by SDS-PAGE (10%). Proteins separated on the gel were electrotransferred onto a PVDF membrane. The membrane was then blocked with 5% non-fat dry milk powder for 3 h, followed by washing with TBST buffer. Subsequently, the membrane was incubated overnight at 4 °C with the appropriate diluted primary antibody. Following further TBST washes, the membrane was incubated with secondary antibody for 2 h at room temperature. Chemiluminescent detection of protein bands was carried out using an ECL reagent kit in accordance with the manufacturer’s protocol, and band intensities were quantified densitometrically with ImageJ software version 1.54h. The following primary antibodies were used: anti-occludin (rabbit monoclonal, 1:1000), anti-JAM-1 (rabbit monoclonal, 1:1000), anti-Nrf2 (rabbit monoclonal, 1:1000), and anti-β-actin (mouse monoclonal, 1:5000). HRP-conjugated anti-rabbit (1:2000) and anti-mouse (1:2000) secondary antibodies were used. Protein bands were detected using enhanced chemiluminescence (ECL) reagent with exposure times ranging from 1 to 5 min. All Western blot experiments were performed with at least three independent biological replicates.

### 2.14. Gut Microbiome Sequencing

DNA was extracted from fecal samples using the TIANamp Stool DNA Kit (Cat# DP328-02; Tiangen Biotech, Beijing, China) according to the manufacturer’s instructions. The V3–V4 region of the 16S rRNA gene was amplified using the universal primers 341F (5′-CCTACGGGNGGCWGCAG-3′) and 806R (5′-GGACTACNVGGGTWTCTAAT-3′). PCR products were purified, and sequencing libraries were constructed using the TruSeq DNA PCR-Free Sample Preparation Kit (Illumina, San Diego, CA, USA). Sequencing was performed on the Illumina NovaSeq 6000 platform (Illumina, San Diego, CA, USA) using a paired-end strategy. Raw reads were quality-filtered using Vsearch (version 2.15.0), and amplicon sequence variants (ASVs) were inferred using the DADA2 pipeline. Taxonomic assignment was performed using the SILVA database (version 138). Samples were rarefied to a uniform sequencing depth of 50,000 reads per sample to normalize sequencing effort across samples. Negative controls were included in each PCR run to monitor potential contamination. Sequencing data have been deposited in the NCBI Sequence Read Archive (SRA).

After receiving the sample, the DNA is collected, and then the sample is tested for quality, and PCR amplification is carried out after passing the quality check. Select one or several variant regions and design them to amplify with primers. Following PCR amplification, electrophoresis was performed to verify the products. Only those PCR products showing normal positive and negative controls within the same batch were considered qualified. The qualified products were then processed for library preparation and quality control (QC). Libraries that passed QC were subjected to high-throughput sequencing using the Illumina NovaSeq platform, and the resulting sequencing data were used for species identification analysis.

Raw paired-end (PE) data were generated through sequencing on the Illumina NovaSeq 6000 platform. These raw reads underwent splicing and quality filtering to remove low-quality sequences, adapters, and PCR-related errors based on predefined criteria, yielding clean tags (equivalent to raw_tags in the report tables). All processing steps relied on the Vsearch software package (version 2.15.0). The clean tags were then dereplicated, and singleton sequences along with chimeras were filtered out to obtain effective tags suitable for downstream analysis.

Representative sequences of amplicon sequence variants (ASVs) were analyzed using the QIIME2 training classifier, which provided species annotation results for each representative sequence. For each sample, QIIME2 was employed to statistically summarize community composition across all taxonomic ranks, including domain, phylum, class, order, family, genus, and species.

For alpha diversity analysis, the command qiime diversity alpha was employed to calculate the Simpson, Ace, Shannon, Chao1, and Goods_coverage indices, all within the QIIME2 environment.

Beta diversity analysis was conducted as follows: based on the species annotation results and operational taxonomic unit (OTU) abundance information from all samples, OTUs belonging to the same taxonomic group were merged to generate a species abundance table (Profiling Table). Subsequently, the phylogenetic relationships among OTUs were used to compute Unifrac distances (unweighted Unifrac). Unifrac distance is a metric that estimates the distance between samples by incorporating evolutionary information from microbial sequences within each sample, producing a distance matrix for multiple samples. Finally, the OTU abundance data were further utilized to construct the unweighted Unifrac distance matrix. Finally, the differences between different samples (groups) were found by multivariate statistical methods such as Principal Component Analysis (PCA) and Principal Coordinate Analysis (PCoA). PCoA results were visualized by Emperor and the software platform QIIME 2 software (version 2024.2).

### 2.15. Statistical Analysis

All data are presented as mean ± SD. In vitro experiments were performed using at least three independent biological replicates (each with technical triplicates where applicable). Animal experiments were performed with n = 6 mice per group. Statistical comparisons utilized one-way ANOVA with Tukey’s post hoc test and a *p* < 0.05 was considered statistically significant.

## 3. Results

### 3.1. LSCP-I Is Non-Cytotoxic and Promotes Epithelial Cell Viability

**Figure 1:**
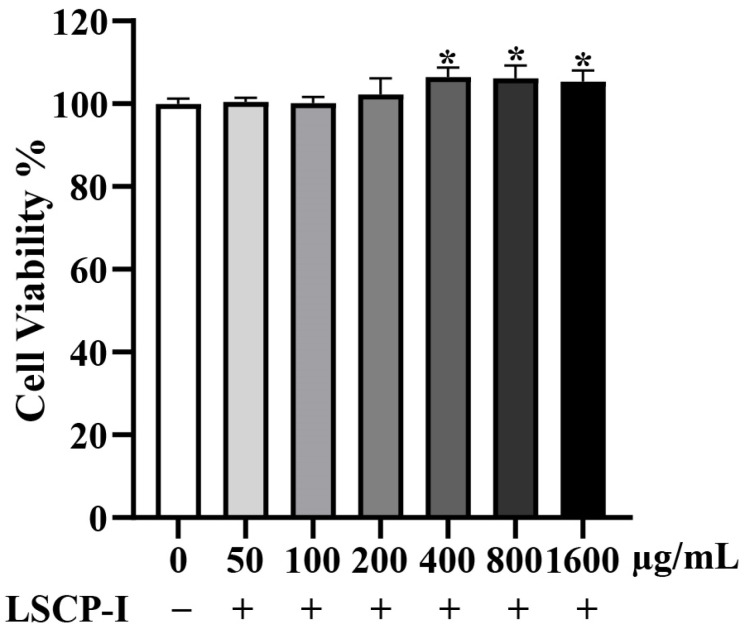
Effects of LSCP-I on the viability of Caco-2 cells. (*) Indicates a *p*-value less than 0.05.

The cytocompatibility of LSCP-I was first evaluated in Caco-2 cells across a concentration range of 50–1600 μg mL^−1^. No cytotoxic effects were observed at 50–200 μg mL^−1^, while a significant (*p* < 0.05) increase in cell viability was detected at ≥400 μg mL^−1^ (Figure 1). These findings indicate that LSCP-I is well tolerated by intestinal epithelial cells and may exert pro-proliferative effects at higher concentrations. Based on these results, subsequent experiments were conducted within this nontoxic and bioactive concentration range.

### 3.2. Establishment of an Inflammatory Epithelial Injury Model via Macrophage-Conditioned Medium

**Figure 2:**
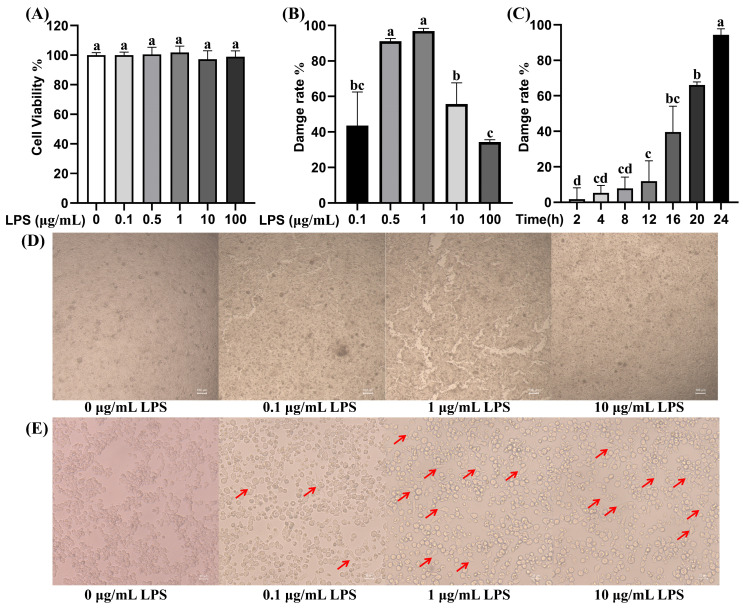
Effects of different inflammatory injury conditions on Caco-2 cell viability. (**A**) Influence of different concentrations of LPS on Caco-2 cell viability. (**B**) Impact of conditioned media from RAW 264.7 cells stimulated with different concentrations of LPS on Caco-2 cell viability. (**C**) Influence of conditioned media from RAW 264.7 cells stimulated with 1 µg/mL LPS for different durations on Caco-2 cell viability. (**D**) Effects of conditioned media from RAW 264.7 cells stimulated with different concentrations of LPS on Caco-2 cell morphology. (**E**) Effects of different concentrations of LPS on RAW 264.7 cell morphology and red arrows indicate RAW cell polarization. Data are expressed as mean ± SD. Different letters or symbols indicate statistically significant differences among groups.

Direct exposure of Caco-2 cells to lipopolysaccharide (LPS) did not significantly (*p* > 0.05) affect cell viability (Figure 2A), indicating limited responsiveness of epithelial monocultures to LPS stimulation alone. To better recapitulate inflammatory microenvironment conditions, a macrophage-mediated injury model was established using RAW 264.7 cells.

LPS stimulation (1 μg mL^−1^) induced morphological changes in RAW 264.7 cells consistent with an inflammatory response, characterized by irregular cell boundaries and altered morphology (Figure 2E). However, because macrophage polarization markers were not comprehensively quantified, the present study does not claim definitive M1 polarization. Conditioned medium derived from these cells induced marked cytotoxicity in Caco-2 cells, with maximal injury observed after 16 h of macrophage stimulation (Figure 2C,D). This condition reduced epithelial viability to approximately 50%, and was therefore selected as the standardized inflammatory injury model for subsequent assays.

### 3.3. LSCP-I Restores Proliferation and Migration of Inflammation-Injured Epithelial Cells

**Figure 3:**
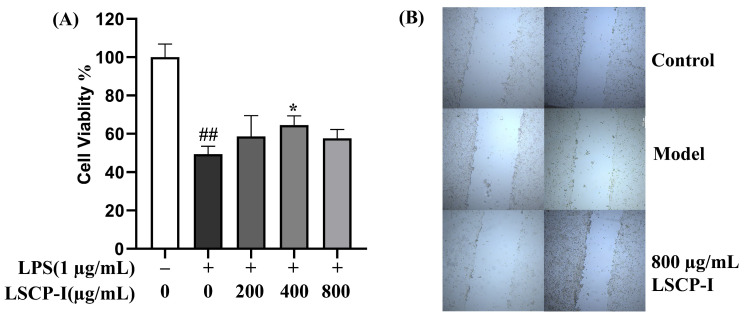
Effects of LSCP-I on proliferation and migration of inflammatory Caco-2 cells: (**A**) cell viability; (**B**) scratch test. (*) represents significance between peptide and model groups (*p* < 0.05). (##) represents significance between control and model groups (*p* < 0.01).

Inflammatory injury markedly impaired epithelial regenerative capacity, as evidenced by reduced viability and diminished migratory activity of Caco-2 cells. Treatment with LSCP-I significantly improved cell viability compared with the injured model group (Figure 3A).

Consistently, scratch wound assays demonstrated that inflammatory conditions suppressed epithelial migration, resulting in incomplete wound closure. LSCP-I treatment restored migratory capacity, promoting wound closure in a concentration-dependent manner (Figure 3B). These results indicate that LSCP-I supports epithelial restitution under inflammatory stress conditions.

### 3.4. LSCP-I Preserves Epithelial Barrier Function Under Inflammatory Stress

**Figure 4:**
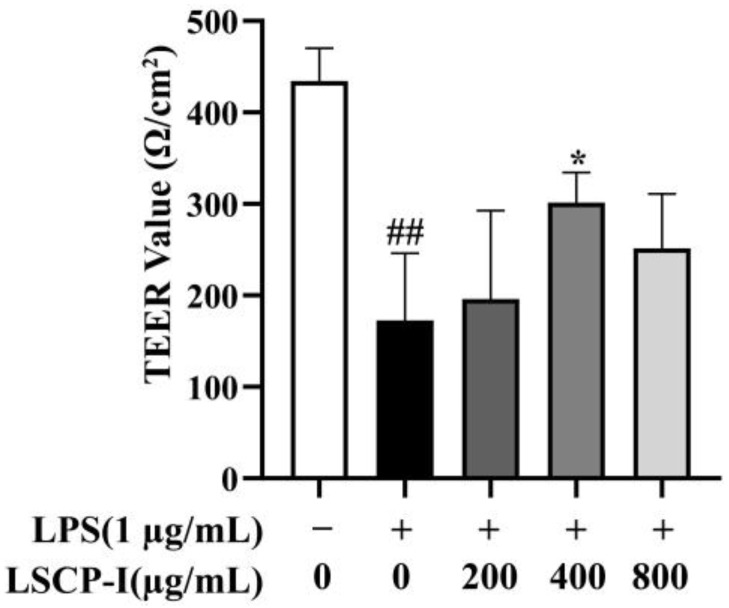
The influence of LSCP-I on the barrier function of inflammation-damaged Caco-2 cells. (*) represents significance between peptide and model groups (*p* < 0.05). (##) represents significance between control and model groups (*p* < 0.01).

Barrier integrity was assessed using transepithelial electrical resistance (TEER) in a transwell-based Caco-2 monolayer system. Inflammatory injury induced by macrophage-conditioned medium significantly reduced TEER values, indicating compromised barrier integrity and increased permeability (Figure 4). LSCP-I treatment significantly attenuated the decline in TEER, partially improving barrier function, with the strongest effect being observed at 400 μg/mL, although TEER values did not fully return to control levels. These findings demonstrate that LSCP-I protects epithelial integrity by maintaining tight junction functionality under inflammatory conditions.

### 3.5. LSCP-I Attenuates Oxidative Stress in Inflammation-Injured Epithelial Cells

**Figure 5:**
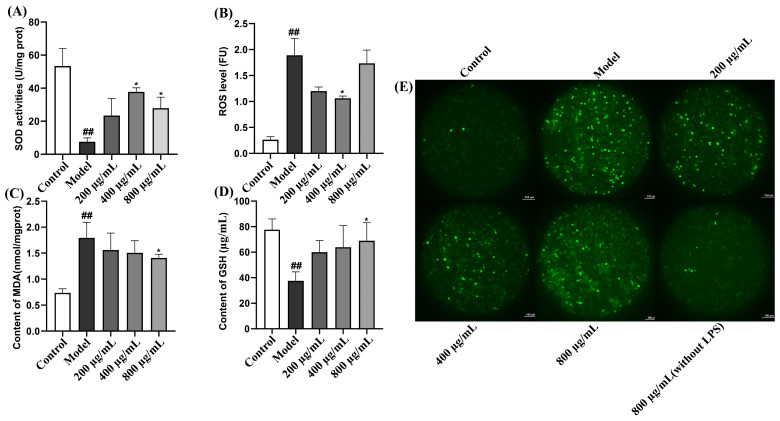
Effect of LSCP-I on the oxidative stress levels of Caco-2 cells under inflammatory injury. (**A**) SOD levels. (**B**) ROS fluorescence intensity. (**C**) MDA levels. (**D**) GSH levels. (**E**) Intracellular ROS levels in Caco-2 cells. (*) represents significance between peptide and model groups (*p* < 0.05). (##) represents significance between control and model groups (*p* < 0.01).

Inflammatory injury was associated with pronounced oxidative stress, characterized by elevated intracellular ROS and malondialdehyde (MDA) levels, alongside decreased antioxidant capacity (Figure 5). Specifically, reduced glutathione (GSH) levels and superoxide dismutase (SOD) activity were significantly (*p* < 0.01) diminished in the model group.

LSCP-I treatment significantly (*p* < 0.05) reduced ROS accumulation and lipid peroxidation, as evidenced by decreased MDA levels. Concurrently, GSH content and SOD activity were restored (Figure 5A–E). These data indicate that LSCP-I effectively re-establishes redox homeostasis in epithelial cells subjected to inflammatory damage.

### 3.6. LSCP-I Alleviates Clinical Symptoms of DSS-Induced Colitis

**Figure 6:**
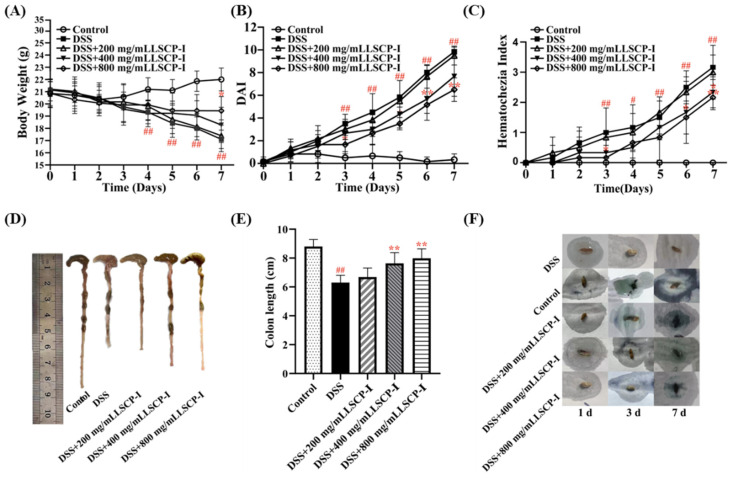
Effects of LSCP-I on body weight, disease index, stool blood and colon length in UC mice: (**A**) changes in body weight; (**B**) changes in DAI; (**C**) stool blood index; (**D**) Colon tissue photos; (**E**) colon length of mice; (**F**) fecal occult blood test results. Data are expressed as mean ± SD. Different letters or symbols indicate statistically significant differences among groups. * *p* < 0.05 and ** *p* < 0.01 versus the control group; # *p* < 0.05 and ## *p* < 0.01 versus the inflammatory model group. Statistical analysis was performed using one-way ANOVA followed by Tukey’s post hoc test.

The therapeutic potential of LSCP-I was assessed in vivo using a mouse model of colitis induced by dextran sulfate sodium (DSS). DSS administration resulted in progressive body weight loss (81.00% of the initial body weight after 7 days of experiment), increased disease activity index (DAI), visible rectal bleeding (Figure 6B,C), and shortening of colon length to 6.31 cm (Figure 6D).

Administration of LSCP-I improved selected colitis-related parameters, with variable responses across doses. Mice treated with the high dose of LSCP-I showed markedly less body weight reduction and lower disease activity index (DAI) scores relative to the DSS group. Moreover, LSCP-I treatment significantly counteracted DSS-induced colon shortening, suggesting a reduction in colonic inflammation (Figure 6E).

### 3.7. LSCP-I Improves Colonic Histopathology

**Figure 7:**
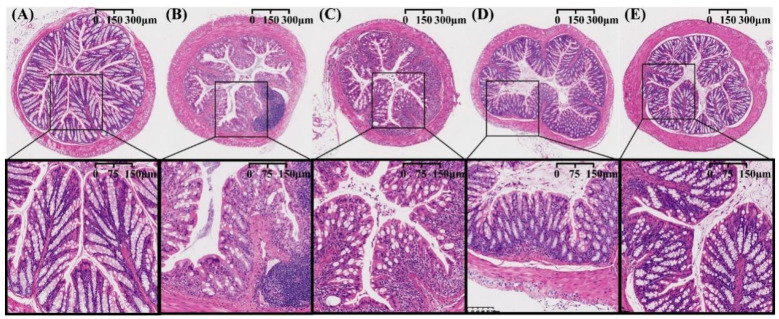
The effect of LSCP-I on the histopathology of colonic tissues in UC mice. (**A**) Control group, (**B**) model group, (**C**) low-dose group (200 mg/mL), (**D**) middle-dose group (400 mg/mL), and (**E**) high-dose group (800 mg/mL).

Histological analysis revealed that DSS exposure caused severe mucosal damage, including epithelial erosion, crypt loss, goblet cell depletion, and extensive inflammatory cell infiltration (Figure 7B).

In contrast, LSCP-I treatment markedly improved colonic architecture. Medium- and high-dose groups exhibited partial restoration of crypt structure, increased goblet cell presence, and reduced inflammatory infiltration (Figure 7D,E). These findings confirm that LSCP-I alleviates structural damage to the intestinal mucosa.

### 3.8. LSCP-I Reduces Intestinal Permeability in Colitis

**Figure 8:**
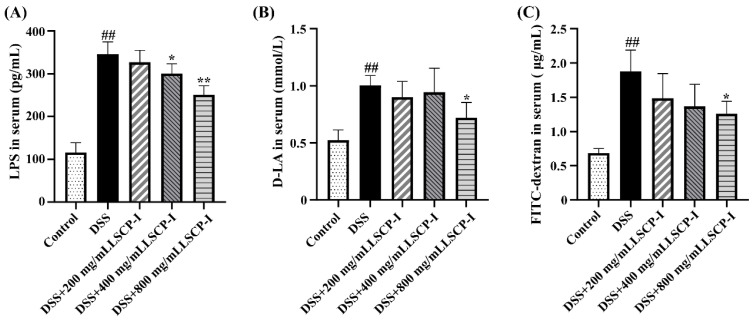
Effects of LSCP-I on intestinal permeability in UC mice: (**A**) LPS level in serum; (**B**) D-LAC level in serum; (**C**) FITC-dextran in serum. (*) represents significance between peptide and model groups (*p* < 0.05). (**) represents significance between peptide and model groups. (##) represents significance between control and model groups *p*-value of *p* < 0.01.

Markers of intestinal permeability were significantly elevated in DSS-treated mice, including serum lipopolysaccharide (LPS), D-lactate (D-LA), and FITC-dextran (Fluorescein isothiocyanate-labeled dextran) levels (Figure 8), indicating disruption of the intestinal barrier.

High-dose LSCP-I significantly reduced all three markers (LPS: *p* < 0.01, D-LA: *p* < 0.05, FITC-dextran: *p* < 0.05), indicating partial improvement but not complete normalization of barrier function. Quantitatively, LSCP-I reduced serum LPS (from 346.23 pg/mL to 251.23 pg/mL), D-LA (from 1 mmol/L to 0.72 mmol/L), and FITC-dextran levels (from 1.87 μg/mL to 1.26 μg/mL) toward control values, confirming improved mucosal barrier function.

### 3.9. LSCP-I Modulates Inflammatory Cytokine Profiles

**Figure 9:**
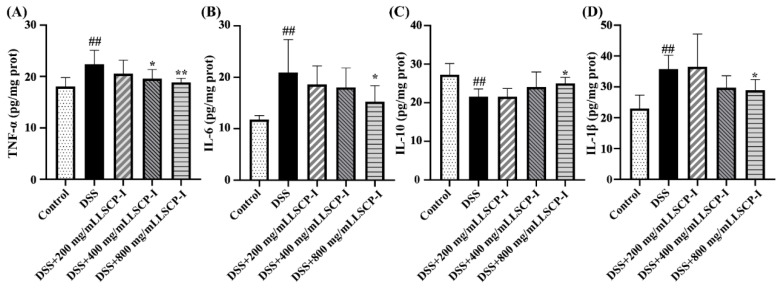
Effects of LSCP-I on the levels of inflammatory factors in colon tissue of UC mice: (**A**) TNF-α inflammatory factor; (**B**) IL-6 inflammatory factor; (**C**) IL-10 inflammatory factor; (**D**) IL-1β inflammatory factor. (*) represents significance between peptide and model groups (*p* < 0.05) and (**) represent a *p*-value of *p* < 0.01. (##) represents significance between control and model groups *p*-value of *p* < 0.01.

DSS treatment triggered a robust inflammatory response, marked by significant (*p* < 0.05) increases in pro-inflammatory cytokines TNF-α (23.89%), IL-6 (77.20%), and IL-1β (55.57%), accompanied by a 20.76% decrease in the anti-inflammatory cytokine IL-10 (Figure 9).

LSCP-I administration dose-dependently suppressed pro-inflammatory cytokine concentrations while simultaneously reinstating IL-10 expression. These findings suggest that LSCP-I helps re-establish immune homeostasis in the intestine under inflammatory conditions.

### 3.10. LSCP-I Enhances Antioxidant Capacity in Colonic Tissue

**Figure 10:**
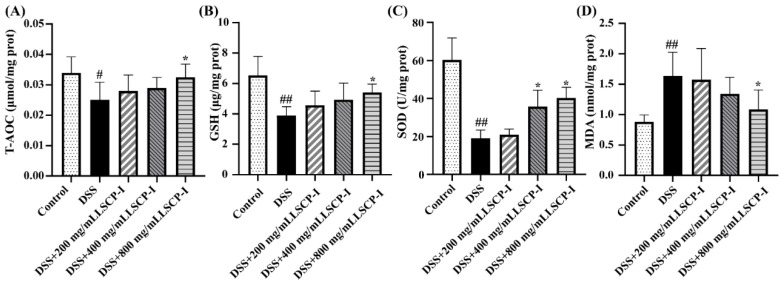
Effects of LSCP-I on antioxidant capacity in colon tissue: (**A**) T-AOC level; (**B**) GSH level; (**C**) SOD activity; (**D**) MDA content. (*) represents significance between peptide and model groups (*p* < 0.05). (#) represents significance between control and model groups (*p* < 0.05) and (##) represent a *p*-value of *p* < 0.01.

DSS-treated mice exhibited a marked rise in colonic oxidative stress, evidenced by a 107.60% increase in MDA levels, along with reductions in total antioxidant capacity (T-AOC), GSH content, and SOD activity (Figure 10).

LSCP-I treatment significantly reversed these alterations. A high dose of LSCP-I reduced lipid peroxidation while restoring antioxidant enzyme activity and redox balance, confirming its in vivo antioxidant efficacy.

### 3.11. LSCP-I Upregulates Nrf2 Signaling and Tight Junction Proteins

**Figure 11:**
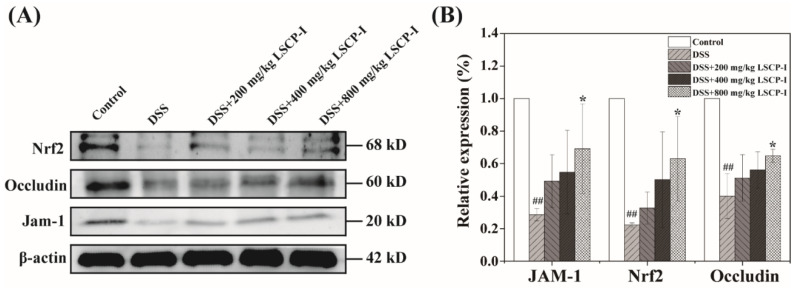
Effects of LSCP-I on the expression levels of Nrf2 and TJs: (**A**) Western blot of Nrf2 and the tight junction markers JAM-1 and Occludin in colonic tissue; (**B**) Densitometric quantification of band intensities of western blot. The experimental groups shown are: Control, DSS-induced colitis, and DSS plus LSCP-1 at low, medium, and high doses (200, 400, 800 mg/kg). Data are expressed as mean ± SD. Different symbols indicate statistically significant differences among groups. (*) represents significance between peptide and model groups (*p* < 0.05). (##) represent a represents significance between control and model groups a *p*-value of *p* < 0.01.

To explore the molecular basis of LSCP-I-mediated protection, the expression of Nrf2 and tight junction proteins was assessed. Exposure to DSS led to a marked reduction in the expression levels of Nrf2 and the tight junction proteins Occludin and JAM-1 (Figure 11).

LSCP-I treatment restored the expression of Nrf2 and tight junction proteins, particularly at higher doses. These findings suggest that LSCP-I enhances intestinal barrier integrity through activation of antioxidant signaling and preservation of junctional complexes.

### 3.12. LSCP-I Partially Restores Gut Microbiota Composition

**Figure 12:**
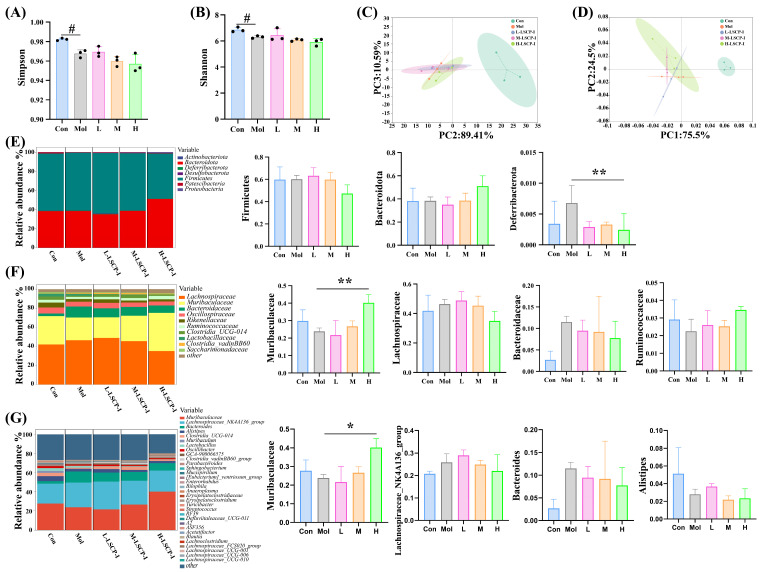
Effects of LSCP-I on the gut microbiota composition in mice: (**A**) Alpha diversity analysis based on the Simpson index. (**B**) Alpha diversity analysis based on the Shannon index. (**C**) Beta diversity inter-group PCA based on Euclidean distance. (**D**) Beta diversity weighted PCoA results. (**E**) Microbial composition at the phylum level. (**F**) Microbial composition at the family level. (**G**) Microbial composition at the genus level. Data are expressed as mean ± SD. Different symbols indicate statistically significant differences among groups. (*) represents significance between peptide and model groups (*p* < 0.05) and (**) represent a *p*-value of *p* < 0.01. (#) represents significance between control and model groups (*p* < 0.05).

DSS-induced colitis significantly altered gut microbiota structure, reducing α-diversity and shifting community composition (Figure 12). Principal Coordinate Analysis demonstrated clear separation between the control and DSS groups, indicating dysbiosis.

LSCP-I treatment did not significantly alter α-diversity indices but shifted microbial composition toward a profile closer to that of healthy controls. At the phylum level, LSCP-I decreased the relative abundance of *Deferribacterota*. At the family and genus levels, LSCP-I significantly enriched the population of *Muribaculaceae*, a microbial taxon linked to the maintenance of intestinal homeostasis. These findings suggest that LSCP-I contributes to the restoration of microbial balance, although its primary protective effects appear to be mediated through host-centered mechanisms.

## 4. Discussion

The present study establishes LSCP-I as a functionally active peptide fraction capable of restoring intestinal homeostasis under inflammatory stress, integrating epithelial repair, oxidative regulation, and host–microbiota interactions. The absence of cytotoxicity and the promotion of epithelial proliferation observed in Caco-2 cells are consistent with emerging evidence that low-molecular-weight food-derived peptides exert trophic effects on intestinal epithelial cells, enhancing viability and regeneration capacity under stress conditions [34,35,36]. Similar proliferative responses have been reported for dietary peptides that act through redox-sensitive signaling and growth-related pathways, supporting the concept that bioactive peptides function not merely as nutrients but as regulators of epithelial renewal [24,35].

A key methodological strength of this work lies in the use of macrophage-conditioned media to model epithelial injury, which more closely reflects the paracrine inflammatory microenvironment than direct lipopolysaccharide exposure. The inability of LPS alone to induce cytotoxicity in Caco-2 cells, contrasted with the pronounced damage mediated by M1-polarized RAW 264.7 cells, aligns with current understanding that epithelial dysfunction in colitis is largely driven by cytokine networks rather than direct endotoxin toxicity [20,21,37]. This reinforces the relevance of the experimental model and situates LSCP-I within a physiologically meaningful inflammatory context.

LSCP-I significantly (*p* < 0.05) improved epithelial proliferation and migration, two processes essential for mucosal restitution. Impaired epithelial migration is a hallmark of ulcerative colitis and contributes to persistent barrier defects. The restoration of scratch closure observed here suggests that LSCP-I enhances epithelial restitution dynamics, potentially via modulation of cytoskeletal remodeling and cell–matrix interactions. Comparable findings have been reported for food-derived peptides that accelerate epithelial repair by activating signaling pathways linked to barrier regeneration [8,38,39]. The exclusion of peptides above 3 kDa in LSCP-I may partly explain its protective effects compared with the broader molecular weight preparation (0.5–5 kDa) used by Li et al. [40], although direct comparative studies are needed to confirm this hypothesis.

Barrier integrity is central to intestinal health, and LSCP-I effectively preserved transepithelial resistance under inflammatory conditions. The decline in TEER observed in the model group reflects disruption of tight junction architecture, a defining feature of colitis pathology. The restoration of barrier function by LSCP-I corroborates prior observations that dietary peptides enhance tight junction protein expression and reduce paracellular permeability in inflamed epithelium [11,15,41]. This effect is particularly relevant in the context of functional foods, where barrier-protective ingredients are increasingly targeted for gut health applications.

Oxidative stress emerged as a central mechanism underlying epithelial injury, as evidenced by elevated ROS and MDA levels and reduced antioxidant defenses. LSCP-I markedly reversed these changes, restoring SOD activity and GSH levels. This antioxidant effect is mechanistically significant (*p* < 0.05), as oxidative stress not only damages cellular components but also amplifies inflammatory signaling cascades. Activation of endogenous antioxidant systems, particularly the Keap1-Nrf2 axis, has been widely reported as a key mechanism through which bioactive peptides mitigate intestinal inflammation [23,26,42]. The upregulation of Nrf2 observed in this study provides strong mechanistic support for LSCP-I-mediated redox regulation.

The in vivo findings further corroborate the protective role of LSCP-I. Improvements in body weight, disease activity index, and colon length collectively indicate attenuation of DSS-induced colitis severity. These outcomes are consistent with established indicators of therapeutic efficacy in colitis models and have been similarly reported for other food-derived peptides with anti-inflammatory properties [8,16,19]. Importantly, the histological recovery of goblet cells and crypt architecture suggests that LSCP-I not only reduces inflammation but also supports mucosal regeneration.

Intestinal permeability is a critical determinant of disease progression in colitis. The reduction in circulating LPS, D-lactate, and FITC-dextran following LSCP-I treatment indicates restoration of barrier integrity at the systemic level. These biomarkers are widely recognized indicators of epithelial leakage, and their normalization reflects improved mucosal sealing. Similar permeability-reducing effects have been observed in studies where dietary peptides reinforce tight junctions and limit endotoxin translocation [11,24,35].

The anti-inflammatory effects of LSCP-I are evidenced by the downregulation of TNF-α, IL-6, and IL-1β, alongside the restoration of IL-10 levels. This rebalancing of cytokine profiles is critical for resolving intestinal inflammation and preventing chronic tissue damage. Future studies should evaluate macrophage activation using molecular markers such as iNOS, CD86, TNF-α, IL-6, IL-1β, Arg-1, and CD206 to determine whether LSCP-I directly modulates macrophage polarization. The observed dose-dependent response further supports a direct bioactive role of LSCP-I. Comparable cytokine modulation patterns have been reported for peptide-based interventions targeting colitis, highlighting their capacity to regulate immune responses at the mucosal interface [8,11,37].

At the molecular level, the coordinated upregulation of Nrf2 and tight junction proteins suggests a dual mechanism of action: enhancement of antioxidant defenses and stabilization of epithelial structure. This coupling of redox signaling with barrier maintenance is increasingly recognized as a central axis in gut health, where Nrf2 activation promotes both cytoprotection and tight junction integrity [15,23,43,44]. The present findings position LSCP-I within this mechanistic framework, linking molecular signaling to functional outcomes.

Finally, modulation of gut microbiota represents an additional layer of LSCP-I activity. Although alpha diversity was not significantly altered, the shift in community structure toward a healthier profile characterized by reduced *Deferribacterota* and increased *Muribaculaceae* suggests selective ecological modulation rather than broad-spectrum restructuring. Such targeted microbiota shifts are increasingly associated with improved metabolic and immune outcomes in colitis models [45,46,47]. The enrichment of beneficial taxa may further contribute to barrier reinforcement and anti-inflammatory effects through microbial metabolite production.

The protective effects of LSCP-I observed in the present study stand in contrast to a recent report by Li et al. [40], who demonstrated that a collagen peptide preparation derived from Walleye pollock (*Theragra chalcogramma*) skin significantly aggravated DSS-induced colitis in mice, as evidenced by increased weight loss, higher mortality, more severe histological damage, elevated pro-inflammatory cytokines (TNF-α, IL-6, IL-1β), enhanced M1 macrophage polarization, activation of NF-κB signaling, and further disruption of gut microbiota composition [40]. This discrepancy is noteworthy and warrants careful consideration, as both studies investigated marine-derived collagen peptides in the DSS-induced colitis model, yet yielded opposing biological outcomes.

Several factors may explain these contrasting findings. First, the biological source and tissue origin differ substantially: LSCP-I was derived from *Lutjanus erythropterus* scales, whereas Li et al. [40] used Walleye pollock skin. Tissue-specific differences in collagen structure, amino acid composition, hydroxyproline content, and embedded bioactive peptide sequences may profoundly influence biological activity [28]. Second, the molecular weight distribution differs markedly: LSCP-I was enriched as a <3 kDa fraction, whereas Li et al. [40] used a broader preparation ranging from 0.5 to 5 kDa. Low-molecular-weight peptide fractions (<3 kDa) are known to exhibit enhanced intestinal absorption, cellular accessibility, and bioavailability compared with higher-molecular-weight populations, which may lead to differential engagement with intracellular signaling pathways [17]. Third, the signaling pathways engaged appear divergent: LSCP-I activated the Nrf2-mediated antioxidant response, whereas Li et al. [40] reported NF-κB activation and M1 macrophage polarization. This suggests that specific peptide sequences and molecular weight profiles may preferentially activate anti-inflammatory/cytoprotective pathways (Nrf2) versus pro-inflammatory pathways (NF-κB), depending on their structural characteristics and cellular interactions. Fourth, differences in DSS concentration (3% in our study vs. 2.5% in Li et al. [40]), treatment duration, dosing regimen, gut microbiota baseline, and mouse genetic background may also contribute to the divergent outcomes [48,49].

Importantly, the present findings should not be interpreted as evidence that all marine collagen peptides are beneficial for intestinal inflammation. Rather, the contrasting outcomes highlight that the biological effects of marine collagen-derived preparations are highly dependent on source species, tissue origin, molecular weight distribution, peptide sequence profile, fractionation strategy, and immune–metabolic context. Thus, marine collagen peptides should be evaluated as source-specific and preparation-specific bioactive fractions rather than as a uniform class of functional ingredients. The exclusion of peptides above 3 kDa in LSCP-I may partly explain its protective effects compared with the broader molecular weight preparation used by Li et al. [40], although direct comparative studies are needed to confirm this hypothesis.

These contrasting findings also underscore the importance of rigorous physicochemical characterization of marine peptide preparations, including molecular weight distribution, amino acid composition, and peptide sequencing, to enable meaningful cross-study comparisons and to establish reliable structure–activity relationships. Future studies should systematically compare collagen peptides from different species, tissues, and molecular weight fractions under standardized colitis models to delineate the determinants of beneficial versus detrimental effects.

Collectively, these findings support a multifactorial mode of action for LSCP-I, encompassing epithelial regeneration, barrier stabilization, oxidative stress attenuation, immune modulation, and microbiota regulation. This integrative functionality aligns with current paradigms in food science that emphasize the development of multifunctional bioactive ingredients for gut health. From a translational perspective, LSCP-I represents a promising candidate for incorporation into functional foods or nutraceutical formulations targeting inflammatory bowel conditions.

## 5. Limitations

The present study provides evidence that LSCP-I protects intestinal epithelial integrity, modulates oxidative stress, and alleviates DSS-induced colitis, but several limitations should be acknowledged. Although sequential ultrafiltration was used to enrich the <3 kDa peptide fraction, this approach does not provide complete peptide sequence-level identification. Therefore, LSCP-I should be considered a low-molecular-weight collagen peptide-enriched fraction. Further LC-MS/MS peptide sequencing, amino acid composition analysis, and molecular weight distribution profiling are required to identify the major bioactive peptide sequences and establish structure–activity relationships. Second, the study was conducted using cellular and murine models; therefore, the findings cannot be directly extrapolated to human ulcerative colitis without clinical validation. Third, although the DSS-induced colitis model is widely used to mimic epithelial injury and intestinal inflammation, it does not fully reproduce the chronic and multifactorial nature of human inflammatory bowel disease. Fourth, the present study did not include a clinically established positive control such as mesalazine. Finally, future studies should include positive controls, peptide sequence identification, dose-translation analysis, safety assessment, and human intervention trials.

## 6. Clinical Translation and Potential Delivery Strategies

The protective effects of LSCP-I observed in cellular and DSS-induced colitis models suggest its potential application as a marine-derived functional ingredient for intestinal health. For clinical translation, LSCP-I could be developed as a nutraceutical supplement, functional food ingredient, or adjunct dietary intervention aimed at supporting intestinal barrier integrity and redox balance. Possible delivery formats include oral capsules, sachet powders, functional beverages, peptide-fortified foods, and enteric-coated formulations designed to improve gastrointestinal stability and targeted intestinal release. Encapsulation technologies, including microencapsulation, nanoencapsulation, and biopolymer-based delivery systems, may further enhance peptide stability during digestion and improve bioavailability [19,35]. However, before human application, additional studies are required to confirm peptide composition, gastrointestinal stability, absorption profile, safety, optimal dosage, and efficacy in clinical populations with ulcerative colitis or intestinal barrier dysfunction. Dose-translation studies from murine models to human equivalents are also needed to establish appropriate intake levels for functional food or nutraceutical applications.

## 7. Conclusions

In conclusion, LSCP-I, a low-molecular-weight peptide-enriched fraction derived from *Lutjanus erythropterus* scales, partially alleviated DSS-induced intestinal injury and improved selected markers related to oxidative stress, barrier integrity, inflammation, and gut microbiota composition. These findings suggest potential for LSCP-I as a source-specific marine peptide fraction for intestinal health applications. In vitro, LSCP-I enhanced epithelial proliferation and migration, preserved barrier integrity, and mitigated oxidative stress in macrophage-conditioned Caco-2 cells. In vivo, it attenuated clinical and histopathological manifestations of DSS-induced colitis, reduced intestinal permeability, rebalanced pro- and anti-inflammatory cytokines, strengthened antioxidant defenses, and partially restored gut microbiota composition toward homeostasis. Mechanistically, these effects were mediated via activation of the Nrf2 antioxidant pathway and maintenance of tight junction protein expression, directly linking redox balance to barrier function. Collectively, these findings suggest that LSCP-I may reinforce intestinal barrier function, support redox homeostasis, and modulate host–microbiota interactions in the DSS-induced colitis model. However, the effects should not be generalized to all marine collagen peptides, as biological outcomes appear to depend on peptide source, molecular-weight distribution, sequence profile, fractionation method, dose, microbiota context, and immune status. Further peptide characterization, positive-control comparisons, safety assessment, and human studies are required before clinical or functional food translation.

## Figures and Tables

**Table 1:** Criteria for disease activity index (DAI) scoring in the DSS-induced colitis.

S. No.	Weight Loss	Fecal Morphology	Blood in the Stool
0	0	The stool is dry and off-white or brownish	Negative
1	1~5%	The stool is slightly moist and elastic	Weak positive
2	6~10%	The feces are sticky and deformed	Positive
3	11~20	The stool is highly viscous; it is too soft to pick up	Strong positive
4	>20%	Water sample stool	Blood in the stool is visible to the naked eye

## Data Availability

The original contributions presented in this study are included in the article. Further inquiries can be directed to the corresponding author.
